# Sleep Disturbances and Obstructive Sleep Apnea in Children and Adolescents with Cerebral Palsy: An Observational Study

**DOI:** 10.3390/neurolint17070101

**Published:** 2025-06-30

**Authors:** Isabella Meneses da Silva, Maria Clara Helena do Couto, Sanseray da Silveira Cruz-Machado, Leticia Monteiro de Andrade, Ana Elisa Zuliani Stroppa Marques, Celia Maria Giacheti, Cristiane Rodrigues Pedroni, Luciana Pinato

**Affiliations:** 1 Undergraduate Program in Speech, Language and Hearing Sciences, Faculty of Philosophy and Sciences, São Paulo State University (UNESP), Campus Marilia 17525-900, Brazil; isabella.meneses@unesp.br (I.M.d.S.); lm.andrade@unesp.br (L.M.d.A.); 2 Graduate Program in Health Sciences and Human Communication, Faculty of Philosophy and Sciences, São Paulo State University (UNESP), Campus Marilia 17525-900, Brazil; clara.couto@unesp.br (M.C.H.d.C.); sanseray@hotmail.com (S.d.S.C.-M.); c.giacheti@unesp.br (C.M.G.); cristiane.pedroni@unesp.br (C.R.P.); 3 Department of Physiotherapy and Occupational Therapy, Faculty of Philosophy and Sciences, São Paulo State University (UNESP), Campus Marilia 17525-900, Brazil; ana.stroppa@unesp.br; 4 Department of Speech, Language and Hearing Sciences, Faculty of Philosophy and Sciences, São Paulo State University (UNESP), Campus Marilia 17525-900, Brazil

**Keywords:** cerebral palsy, sleep apnea syndromes, oximetry, sleep disorders, children

## Abstract

Background/Objectives: Cerebral palsy (CP) is a neurodevelopmental disorder associated with sleep disturbances, particularly sleep-disordered breathing (SDB), and is often linked to an increased risk of obstructive sleep apnea (OSA). OSA is underdiagnosed in this population due to the lack of standardized methods and limited access to appropriate diagnostic technologies and appropriate equipment. Thus, this study aimed to investigate the presence and severity of sleep disorders, with a particular focus on OSA, in children and adolescents with CP compared to their typically developing peers. Methods: This observational, clinical, and prospective study included 28 children and adolescents with CP and 32 age- and sex-matched typically developing individuals. Sleep disturbances were assessed using the Sleep Disturbance Scale for Children (SDSC) and a high-resolution oximeter plus actigraphy combined with a cloud-based algorithm for the detection of obstructive sleep apnea (Biologix^®^ system), which provided data on oxygen saturation, snoring, movement during sleep, and total sleep time. Results: According to the SDSC, 92% of children and adolescents with CP presented scores indicative of sleep disturbances, compared to 31% of typically developing individuals. SDB was the most prevalent subtype (64%) and overnight oximetry revealed that 100% of the CP group presented oxygen desaturation index (ODI) values consistent with a diagnosis of OSA. The CP group also exhibited significantly lower mean SpO_2_, longer snoring duration, shorter total sleep time, and prolonged sleep latency compared to the typically developing group. Conclusions: Children and adolescents with cerebral palsy (CP) exhibit a high prevalence of sleep disturbances, with increasing evidence indicating a significant occurrence of sleep-disordered breathing (SDB), particularly obstructive sleep apnea (OSA).

## 1. Introduction

Cerebral palsy (CP) is characterized by permanent and non-progressive neurological alterations caused by pre-, peri-, or postnatal factors that may affect the developing brain, resulting in persistent disorders of muscle tone, posture, and movement at different levels [1,2]. It is considered the most common motor disorder in childhood, affecting 2 to 3 per 1000 live births [3]. Since brain development alterations occur due to multiple causes, and may manifest in various clinical presentations and severities, CP has been classified into categories based on movement disorder type, affected area, and severity level [4].

Although motor impairment is the most prominent feature, sensory, perceptual, cognitive, and behavioral disorders are also present [5]. The most frequent coexisting impairments include pain, intellectual disability, hip dysplasia, speech problems, epilepsy, behavioral disorders, bladder control issues, blindness, and deafness [6]. Individuals with CP frequently exhibit chronobiological dysfunctions, including abnormal rhythm of melatonin and sleep disturbances, although putative mechanisms remain unclear [7].

The prevalence of sleep disorders in individuals with cerebral palsy (CP) has been reported to range from 47% to 60%, based on subjective assessments using questionnaires and rating scales, with sleep-disordered breathing (SDB) identified as the most common subtype [7,8]. Sleep-disordered breathing (SDB), encompassing a continuum from primary snoring to obstructive sleep apnea (OSA), represents a spectrum of clinical conditions marked by upper airway dysfunction during sleep. This dysfunction manifests as snoring and/or increased respiratory effort and is primarily attributed to elevated upper airway resistance and increased pharyngeal collapsibility [9]. OSA, in particular, is characterized by intermittent episodes of upper airway obstruction of varying severity, leading to disrupted sleep architecture and alterations in gas exchange [10]. These disturbances contribute to both nocturnal and daytime chronodisruption and are associated with long-term adverse health outcomes. Therefore, early recognition and appropriate diagnosis of SDB are critical to preventing or attenuating consequences such as impaired cognitive development, behavioral problems, poor academic performance, heightened cardiovascular risk, reduced quality of life, and increased impacts on healthcare systems through cumulative treatment costs and the management of its long-term health consequences [11].

The general pediatric prevalence of obstructive sleep apnea (OSA) is estimated to range from 1% to 4% [12,13], although these figures vary considerably across the limited number of studies available, likely due to differences in diagnostic criteria, assessment tools, and population characteristics. Pediatric sleep-disordered breathing (SDB), including OSA, may represent a major—yet still under-recognized—public health burden, with long-term consequences for affected individuals, their families, and society. It is highly probable that many cases remain undiagnosed, and even among those identified, a substantial proportion do not receive appropriate treatment [14]. Children with CP are particularly vulnerable to OSA due to impaired neuromuscular control of the upper airway [15,16]. The OSA in this population can significantly impact quality of life and contribute to a range of adverse health outcomes, particularly neurocognitive impairments [17]. In severe, untreated cases, OSA can progress to cardiorespiratory failure and even death [18].

This study aimed to investigate the presence of sleep disorders, with a particular focus on OSA, in a group of children and adolescents diagnosed with CP and to age-matched individuals with typical development. While including typically developing individuals for contextualization, the study focuses on identifying and characterizing the variability and severity in the prevalence and clinical expression of OSA among children and adolescents with CP, which may contribute to a better understanding of their specific vulnerability to sleep-disordered breathing (SDB). The findings also may support the development of targeted screening strategies and early interventions to mitigate the adverse health outcomes associated with untreated OSA in this population.

## 2. Materials and Methods

### 2.1. Study Design

This was a primary, observational, clinical, and transversal study in which eligible research subjects were observed regarding variables related to their characteristics and sleep disturbances and were compared to typically developing children and adolescents (TD). This study was approved by the Ethical Committee of the Sao Paulo State University—Unesp (ethical approval code 6.098.302). All participants and/or their legal guardians were fully informed about the study design and signed the Free and Informed Consent/Assent Forms, in accordance with Resolution 466/12 of the Brazilian National Health Council. The procedures were conducted at the Center for Studies in Education and Health or at the individual’s home.

Following the formation of the CP group, the TD group was composed in a matched manner, considering age, sex, and Body Mass Index (BMI). Recruitment of TD children and adolescents, confirmed by caregiver reports and the absence of known neurological or motor disorders, was conducted through public schools in the same municipality as the participants with CP. An educational flyer containing a brief description of the research and an invitation to participate was distributed to families via the school network.

### 2.2. Sample Size Calculation

Due to the limited number of studies assessing OSA in individuals with cerebral palsy (CP), we based our sample size calculation on previous research employing a similar methodological approach, using data from typically developing children and children with autism spectrum disorder within the same age range [19]. The minimum required sample size was estimated using the formula proposed by [20] for case–control studies: The minimum required sample size was estimated using the formula proposed by [20] for case–control studies: N = (r + 1/r) × ((SD^2^ × (Z_β_ + Z_a_/_2_)^2^)/d^2^), where r is the ratio of controls to cases (assumed to be 1), SD is the standard deviation of the main outcome variable (total sleep time, estimated at 0.9 h), Z_a_/_2_ corresponds to the confidence level (1.96 for α = 0.05), Z_β_ represents the statistical power (1.28 for 90% power), and d is the expected difference between group means (–1.37 h). Based on these parameters, the estimated minimum sample size was nine participants per group. We also calculated the minimum sample size using an alternative outcome: the total score from the Sleep Disturbance Scale for Children (SDSC). For this analysis, we used previously published data comparing children who stutter with TD children, assuming a 1:1 group ratio, a standard deviation of 6.61, and an expected difference between group means of 13.36 [21]. In this case, the minimum sample size was estimated at six participants per group. In the present study, we assessed the presence of sleep disorders in 32 TD children and 28 individuals with CP.

### 2.3. Inclusion Criteria

The sample consisted of individuals diagnosed with CP, aged between 4 and 17 years, as well as age- and sex-matched typically developing individuals who composed the TD group. Individuals with associated syndromes or genetic alterations were excluded from the study as well as children and adolescents with a medical diagnosis of any type of severe malformation, severe visual impairment or blindness, or other congenital anomalies (physical or mental), and those using melatonin or medications that affect sympathetic neurotransmission (such as beta-adrenergic receptor blockers or reuptake inhibitors) or any drug known to interfere with melatonin biosynthesis [19].

### 2.4. Study Population

Samples were descriptively, characterized either by absolute frequency (N) or relative percentage frequency (%), in accordance with clinical assessment guidelines recommended by the São Paulo Pediatric Society. Family socioeconomic stratification was determined according to the criteria established by the Brazilian Economic Classification Criteria (Brazilian Economic Classification Criteria (Brazilian Criteria)) [22]. In the CP group, 80% belonged to socioeconomic group DE (classes with lower purchasing power) and 20% to C2 (middle classes), while in the typically developing group, 45% belonged to the socioeconomic group B2 (intermediate classes), 35% to C2, and 20% to DE.

### 2.5. Functional Classification of Cerebral Palsy

Assessing the severity of motor impairments is crucial for determining the functionality of affected limbs and predicting treatment outcomes. Several classification systems are used for this purpose, including the Gross Motor Function Classification System (GMFCS) [4]. The GMFCS is widely used globally for the functional classification of CP. It is a relatively simple scale that evaluates gross motor function based on voluntary movement and the use of assistive devices. The revised version applies to individuals aged 2 to 18 years and categorizes motor function into five levels: the child can walk without assistance, but age-related considerations apply (Level I); the child can perform all motor activities but with limitations in speed, balance, and endurance (Level II); the child walks with handheld mobility devices indoors, requires supervision when climbing stairs, and uses wheeled mobility for long distances (Level III); the child lacks independent mobility, can sit with support, and requires a manual or powered wheelchair for transportation (Level IV); and the child is fully dependent in all settings and has limitations in maintaining an antigravity posture (Level V) [23]. In the present study, this evaluation was performed at the Center for Studies in Education and Health (São Paulo State University) by a trained/specialist physical therapist.

### 2.6. Sleep Disturbance Scale for Children (SDSC)

The Sleep Disturbance Scale for Children was developed by [24] and adapted to Portuguese by [25]. This scale, applicable to individuals aged 3 to 18 years, including children with cerebral palsy, presents 28 questions and screens for the following sleep disturbances: disorders of initiating and maintaining sleep (DIMS), sleep breathing disorders (SBD), disorders of arousal (DA), sleep–wake transition disorders (SWTD), excessive daytime sleepiness (EDS), and sleep hyperhidrosis (SH). Responses were provided by the child’s caregiver. Scoring ranges from 1 to 5 (1 = never, 2 = occasionally, 3 = sometimes, 4 = often, and 5 = always), reflecting the frequency of each symptom over the past six months. The total score provides an overview of specific sleep disorder domains, and at reaching 39 points the presence of a sleep disorder may be considered. The questionnaire also comprises six subscales, each with a cutoff score: sleep initiation and maintenance disorders (DIMSs, cutoff: 21 points); sleep-disordered breathing (SDB, cutoff: 6 points); arousal disorders (AD, cutoff: 11 points); sleep–wake transition disorders (SWTD, cutoff: 23 points); excessive daytime sleepiness (EDS, cutoff: 19 points); and sleep hyperhidrosis (SH, cutoff: 7 points).

### 2.7. Oximetry

Continuous overnight recording of oxygen saturation by a high-resolution oximeter plus actigraphy combined with a cloud-based algorithm for the detection of obstructive sleep apnea can be used to estimate the presence of OSA. This may shorten the diagnostic and therapeutic process for children and adolescents with more severe disease either at home or in the hospital [26,27,28,29,30]. It is an appealing alternative to polysomnography (PSG) due to its cost-effectiveness, its availability in most centers, and its feasibility for quantitative analysis.

In the present study, we employed a high-resolution wireless oximeter (Oxistar™, Biologix Systems S.A., São Paulo, Brazil), capable of assessing obstructive sleep apnea (OSA). The assessment was conducted over a single night in the participant’s home environment, providing a more naturalistic and comfortable setting. The Biologix system consists of a high-resolution wireless pulse oximeter with an integrated accelerometer, connected via Bluetooth to a smartphone application that records snoring. All data is automatically transmitted to a secure cloud-based platform, where a proprietary Biologix algorithm analyzes the information and generates an automated diagnostic report [27,28,29].

The Oxistar™ firmware acquires oxygen saturation data at a sampling rate of 100 Hz with a resolution of 0.1%, enabling precise detection of oxygen desaturation events per hour of valid recording, known as the oxygen desaturation index (ODI). In addition to oximetry, the system provides measurements of heart rate, body movement, sleep estimation, and snoring, which are critical parameters for diagnosing obstructive sleep apnea (OSA) [30].

The Biologix system has been validated for OSA diagnosis in adults against both type 1 and type 3 polysomnography [27,29]. Although it has not yet been validated in the pediatric population, recent studies have demonstrated its feasibility and applicability for OSA detection in children with craniofacial anomalies [30].

The ODI ≥ 3 events/hour was adopted as the threshold to indicate the presence of obstructive sleep apnea (OSA), based on prior research that considers this criterion clinically meaningful in pediatric populations [9,31,32].

Given that this study was conducted with children and adolescents, the quality of the SpO_2_ signal obtained in the current assessments was evaluated by comparing the average SpO_2_ signal quality from this study (mean ± SD: 91.26 ± 9.5%) with data from 12,615 adults of the general population in the Biologix Systems S.A. database, which reports a mean ± SD of 97.15 ± 5.06%. Thus, the recordings obtained in the present study in children and adolescents demonstrated a good signal quality for SpO_2_ detection when compared to adult recordings.

### 2.8. Data Analysis

Data were analyzed using SPSS 24.0 and GraphPad Prism 9.0 software. The normality of the data was assessed using the Shapiro–Wilk test. After testing for normality, results were expressed as median (interquartile range 25–75%) or relative percentage frequency (%). The Chi-square test was applied to compare categorical variables between groups. The Mann–Whitney test was used for between-group comparisons of sleep parameters. The significance level was set at *p* < 0.05.

## 3. Results

The demographic and clinical profiles of participants from both groups are summarized below. Age, sex, and functional motor classifications were evaluated for children with typical development (TD) and those with cerebral palsy (CP). The Gross Motor Function Classification System (GMFCS) was used to assess motor impairment in the CP group. Detailed data are presented in Table 1.

### Sleep Disorders and Objective Sleep Parameters in Individuals with Cerebral Palsy and Typically Developing Individuals

Assessment using the SDSC revealed that 92% of individuals with CP exhibited indications of sleep disorders according to the total scale score. Specifically, 64% showed signs of sleep breathing disorders (SBD), 21% presented with disorders of initiating and maintaining sleep (DIMS), 14% with sleep hyperhidrosis (SH), 7% with excessive daytime sleepiness (EDS), and 4% with sleep–wake transition disorders (SWTD). In the typically developing group, the identified sleep disturbances were DIMS in 13% of the sample, and SH in 3% of the evaluated individuals. Based on the total SDSC score, 31% of typically developing participants showed indications of sleep disturbances (Table 2).

Next, we evaluated the median scores and interquartile ranges from the SDSC for both the CP and TD groups. Children and adolescents with CP exhibited significantly higher total SDSC scores compared to the TD group (*p* < 0.001), indicating a greater overall burden of sleep disturbances. Notably, children and adolescents with CP scored significantly higher in the subscales for DIMS (*p* < 0.001), SDB (*p* < 0.001), SWTD (*p* < 0.05), and SH (*p* < 0.05). No significant differences were observed in the arousal disorders or in the sleep–wake transition subscales. These results highlight significantly compromised sleep quality among children and adolescents with CP, as evidenced by elevated scores across multiple domains of the SDSC, when compared to TD individuals (Table 3).

Regarding the objective analysis of sleep parameters using high-resolution oximetry, the total sleep time in the CP group was 7 (5–8) hours, and sleep latency was 19 (12–23) minutes. In the TD group, total sleep time was 8 (7–8) hours, and sleep latency was 19 (15–31) minutes. No significant differences were found between the groups for these variables. However, the duration of snoring accounted for 27% of total sleep time in the CP group, which was significantly higher than in the TD group (1% of total sleep time; *p* = 0.03) (Table 4).

As for oxygen desaturation parameters, the mean SpO_2_ among individuals with CP was significantly lower than that observed in the TD group, 95% (94–96) vs. 96% (96–97); *p* = 0.006. On average, individuals with CP spent approximately mean ± SD: 5 +/− 0.3% of their total sleep time (TST) with oxygen saturation (SpO_2_) below 90%. The average minimum SpO_2_ value was 84.3 ± 7.3% (mean ± SD) in the CP group, while in the TD group it was 87.2 ± 3.5%; the mean SpO_2_ value was 95.1 ± 1.2% in the CP group, while in the TD group it was 96.0 ± 0.9% (*p* = 0.03); and the average maximum SpO_2_ value was 99.6 ± 0.7% in the CP group, while in the TD group it was 99.8 ± 0.4%. All children and adolescents in the CP group (100%) exhibited an oxygen desaturation index (ODI) indicative of sleep apnea, with a median ODI of 3.6 (3.1–7.8) events per hour (Table 4).

## 4. Discussion

Considering the importance of identifying sleep disorders to guide therapeutic planning in children and adolescents with cerebral palsy (CP), the present study investigated the prevalence and profiles of sleep disturbances in this population using the Sleep Disturbance Scale for Children (SDSC). The findings revealed that 92% of the participants with CP presented scores suggestive of sleep disorders based on the total SDSC score—substantially higher than the 31% observed in a typically developing reference group. Among the sleep disorders, sleep-disordered breathing (SDB) was the most prevalent subtype. However, other domains—such as disorders of initiating and maintaining sleep (DIMS), sleep–wake transition disorders (SWTD), and sleep hyperhidrosis (SH)—were also present and deserve clinical attention.

Previous studies using SDSC have already demonstrated a high prevalence of sleep disturbances in individuals with CP [33,34], with negative impacts not only on the affected individuals but also on the quality of life of their caregivers and families [35]. Notably, a substantial proportion of children and adolescents with CP exhibit symptoms consistent with SDB, as reported in various investigations [7,8,36,37]. Although children and adolescents with neurodevelopmental disorders are generally at increased risk for obstructive sleep apnea syndrome (OSA), this risk appears to be further elevated in those with CP.

In alignment with these findings, the present study identified that 64% of children and adolescents with CP had SDSC scores suggestive of SDB, reinforcing previous evidence [7,8] and highlighting the need for systematic screening and early management of sleep-disordered breathing in this population. Considering the importance of characterizing sleep disorders for therapeutic planning in this population, the present study assessed the presence of sleep disorders, which showed that 92% of individuals with CP presented signs of sleep disorders according to the total score of the scale evaluated by SDSC. This percentage was higher than those of the TD group (31%). Among the sleep disorders, the most frequent in the CP group was SBD, but attention should also be paid others with lower frequency, such as DIMS, SWTD, and SH.

Although the SDSC is widely used in studies involving children and adolescents with cerebral palsy, it is important to acknowledge that this scale was not originally developed for children and adolescents with severe motor and cognitive impairments. Therefore, its results should be interpreted with caution and, whenever possible, complemented by objective sleep assessment tools.

In this regard, the present study further evaluated SBD by using an objective measurement through a high-resolution oximeter plus actigraphy combined with a cloud-based algorithm for the detection of obstructive sleep apnea [29]. Particularly regarding the presence of OSA, the present study showed that 100% of individuals with CP exhibited an oxygen desaturation index (IDO) indicative of OSA. In terms of oxygen desaturation, the CP group had a lower average SpO_2_. Additionally, the duration of snoring was higher than that found in the typically developing group.

Children with CP are at increased risk for sleep-disordered breathing (SDB) and OSA due to several interrelated pathophysiological mechanisms that contribute to upper airway collapse. These include craniofacial abnormalities, altered upper airway tone or morphology, and oropharyngeal dysfunctions such as dysphagia, all of which can compromise airway patency during sleep [38]. Additionally, intellectual disability and impaired neuromuscular control may further exacerbate the risk of respiratory events during sleep. Recognizing these underlying mechanisms highlights the importance of early identification and the need for tailored screening approaches, especially in clinical settings where full polysomnography may not be readily accessible.

Diagnostic methods for sleep-disordered breathing (SDB) in children and adolescents typically involve a combination of clinical assessment, medical history, standardized questionnaires, and objective measurements such as polysomnography. Although some studies have addressed this issue, objective data specifically examining the association between OSAS and cerebral palsy (CP) remain limited. A few investigations have reported elevated apnea indices in children with CP. For example, the authors in [39] found that 49% of children with CP exhibited moderate to severe OSA when assessed via polysomnography. This finding, together with the present study’s results, underscores the heightened vulnerability of this population to SDB [40,41]. It is noteworthy that the present study evaluates both subjective and objective measures related to sleep disorders in PC and compares them to the scores from a TD group.

Sleep-disordered breathing (SDB), including OSA, has significant health consequences, including impaired daytime exhaustion due to excessive sleepiness, as well as increased risk of comorbidities, such as cardiovascular failure, metabolic disorders, and increased risk of mortality. In individuals with cerebral palsy (CP), approximately 34% of deaths are attributed to respiratory causes, and sleep-related breathing disorders play a prominent role as an identifiable risk factor [42]. Timely and accurate diagnosis is therefore essential for effective management and intervention.

In the present study, in addition to oxygen saturation data, the oximetry system enabled the assessment of sleep parameters. The total sleep time observed in the CP group was 7 (5–8) h, which falls below the minimum recommended duration for this age group [43]. Moreover, sleep latency was 19 (12–23) min, exceeding the acceptable median of 15 min [44].

Objective data on sleep parameters are limited in children and adolescents with CP, possibly due to the known limitations of conducting full polysomnography (PSG) in this population. Although PSG is considered the gold standard for OSA diagnosis, it is time-consuming, with high costs, and generally not widely available, particularly in less-resourced areas. In this context, overnight oximetry has emerged as a simpler and potentially more accessible screening tool, offering predictive value in identifying children at high risk for OSA [29]. Nonetheless, a notable strength of our study is the use of a high-resolution oximeter combined with actigraphy and a cloud-based algorithm for the detection of obstructive sleep apnea—a method previously applied in pediatric populations [30], but employed here for the first time to assess the presence of OSA specifically in children and adolescents with CP.

Although this method provides valuable data on key sleep parameters (as total time of sleep and sleep latency), it does not offer the full range of information available through standard in-lab polysomnography, such as electroencephalographic recordings or detailed sleep staging. Compared to other objective measures, such as actigraphy, which has been increasingly used in sleep research, the use of overnight oximetry offers distinct advantages. While actigraphy relies on movement to infer sleep–wake patterns, a major limitation of actigraphy in individuals with CP is their restricted mobility. On the other hand, oximetry directly captures physiological indicators of respiratory events, such as oxygen desaturation, making it a more reliable screening tool for SDB in this context.

### Limitations

One relevant limitation of this study is the lack of heterogeneity within the cerebral palsy (CP) group. Approximately 90% of the participants with CP were classified as GMFCS level V, representing the most severe level of motor impairment. As such, the findings primarily reflect sleep-related characteristics in children with profound functional limitations and may not be generalizable to the broader CP population, particularly those with milder motor involvement. While the focus on a severely affected subgroup provides important insights into a highly vulnerable population, caution should be exercised when extending these results to children across the full spectrum of CP severity.

Although the Sleep Disturbance Scale for Children (SDSC) has been widely used in studies involving children with cerebral palsy (CP) [35,45,46,47], it is important to acknowledge that the scale was originally developed and validated for typically developing children. As such, some items may be challenging to interpret in populations with severe motor and/or cognitive impairments, such as those classified at higher GMFCS levels. Consequently, SDSC results in this context should be interpreted with caution and ideally complemented by objective measures. In this study, the use of high-resolution wireless oximetry provided additional data to support and contextualize the subjective findings. While the SDSC remains a useful screening tool due to its broad coverage of sleep domains and capacity for group comparisons, we emphasize the need for future research to validate or adapt sleep assessment instruments specifically for populations with complex neurodevelopmental profiles.

## 5. Conclusions

Individuals with CP showed a higher score of sleep disorders evaluated by SDSC, particularly a higher frequency of SBD and DIMS, compared to age-matched typically developing individuals. In addition, individuals with CP exhibited oxygen desaturation index values indicative of OSA, reduced time of sleep, and increased sleep latency, evaluated here for the first time through a high-resolution oximeter. Altogether, our findings highlight the clinical utility and feasibility of integrating oximetry-based assessments into sleep evaluations for children and adolescents with CP, particularly in settings where full polysomnography is unavailable or impractical.

## Figures and Tables

**Table 1:** Demographic and clinical characteristics of study participants.

Variables	TD n = 32	PC n = 28
Age, mean ± SD (years)	10.2 ± 1.8	11.3 ± 2.9
Sex (%)		
Female	34	54
Male	66	46
GMFCS (%)		
Level V	Not applicable	89.3
Level III	Not applicable	7.1
Level II	Not applicable	3.6

Legend: Demographic and clinical characteristics of the participants in the typically developing (n = 32) and cerebral palsy (CP) groups (n = 28). Data for age are expressed as mean ± standard deviation (SD). Sex distribution is reported as percentages. In the CP group, functional motor impairment was classified according to the Gross Motor Function Classification System (GMFCS), with percentages indicating the distribution across levels II, III, and V.

**Table 2:** Frequency of sleep disorders in children and adolescents with cerebral palsy (CP) and typically developing (TD) individuals based on the SDSC.

	TD (n = 32)	CP (n = 28)
	%	%
**Total SDSC score**	**31**	92 **
Sleep breathing disorders	13	64
Disorders of initiating and maintaining sleep	0	21
Sleep hyperhidrosis	3	14
Excessive daytime sleepiness	0	7
Sleep–wake transition disorders	0	4
Disorders of arousal	0	0

Legend: The percentage of children and adolescents in each group (CP, n = 28; TD, n = 32) who exceeded the clinical cut-off scores for the total and subscale domains of the Sleep Disturbance Scale for Children (SDSC). Chi-square test was used for statistical analysis. Values are expressed as percentages within each group. “**” indicates a statistically significant difference between groups (*p* < 0.001).

**Table 3:** Sleep Disturbance Scale for Children (SDSC) scores in children and adolescents with cerebral palsy and typically developing individuals (TD).

	TD		CP	
	Median	IQR	Median	IQR
Total SDSC score	36	(33–40)	56	(46–70) **
Disorders of initiating and maintaining sleep	10	(8–12)	15	(12–21) **
Sleep breathing disorders	3	(3–5)	8	(4–11) **
Disorders of arousal	3	(3–3)	3	(3–4)
Sleep–wake transition disorders	8	(6–11)	12	(8–16) *
Disorders of excessive somnolence	6	(5–9)	11	(5–17) *
Sleep hyperhidrosis	2	(2–3)	4	(2–6) *

Legend: Data are presented as median values with interquartile ranges (IQR) (25–75%) for each domain of the SDSC in children and adolescents with cerebral palsy (CP; n = 28) and typically developing children and adolescents (TD, n = 32). Mann–Whitney U test was used for statistical analysis. Higher scores reflect greater sleep disturbance. * *p* < 0.05; ** *p* < 0.001.

**Table 4:** Sleep parameters assessed by high-resolution oximeter plus actigraphy combined with a cloud-based algorithm for the detection of obstructive sleep apnea in children and adolescents with cerebral palsy (CP) and typically developing (TD) individuals.

Parameter	TD	CP
Total sleep time (hours)	8 (7–8)	7 (5–8)
Sleep latency (minutes)	19 (15–31)	19 (12–23)
Snoring duration (% of TST)	1 (0–18)	27 (5–44) *
Mean SpO_2_ (%)	96 (96–97)	95 (94–96) *
Oxygen desaturation index (ODI; events/h)	Not reported	3.6 (3.1–7.8)
Oxygen desaturation < 90% (% of group)	11	22

Legend: Data are presented as median and interquartile range (IQR: 25–75%) unless otherwise specified. TST = total sleep time; SpO_2_ = oxygen saturation; ODI = oxygen desaturation index; Mann–Whitney U test was used for statistical analysis. * Statistically significant difference between groups (*p* < 0.05).

## Data Availability

The data that support the findings of this study are available from the corresponding authors upon reasonable request.
